# Deep long period volcanic earthquakes generated by degassing of volatile-rich basaltic magmas

**DOI:** 10.1038/s41467-020-17759-4

**Published:** 2020-08-06

**Authors:** Oleg Melnik, Vladimir Lyakhovsky, Nikolai M. Shapiro, Natalia Galina, Olga Bergal-Kuvikas

**Affiliations:** 1 Institute of Mechanics, Moscow State University, 1 Michurinskiy prospekt, 119192 Moscow, Russia; 2 Geological Survey of Israel, 32 Yesha’ayahu Leibowitz st, 9692100 Jerusalem, Israel; 3 Institut de Sciences de la Terre, Université Grenoble Alpes, CNRS (UMR5275), CS 40700, 38058 Grenoble Cedex 9, France; 4 Schmidt Institute of Physics of the Earth, Russian Academy of Sciences, Bolshaya Gruzinskaya str., 10-1, 123242 Moscow, Russia; 5 Institute of Volcanology and Seismology, FEB RAS, 9 Piip Boulevard, 683006 Petropavlovsk-Kamchatsky, Russia

**Keywords:** Seismology, Volcanology

## Abstract

Deep long-period (DLP) earthquakes observed beneath active volcanoes are sometimes considered as precursors to eruptions. Their origin remains, however, unclear. Here, we present a possible DLP generating mechanism related to the rapid growth of gas bubbles in response to the slow decompression of over-saturated magma. For certain values of the gas and bubble content, the elastic deformation of surrounding rocks forced by the expanding bubbly magma can be fast enough to generate seismic waves. We show that amplitudes and frequencies of DLP earthquakes observed beneath the Klyuchevskoy volcano (Kamchatka, Russia) can be predicted by our model when considering pressure changes of ~10^7^ Pa in a volume of ~10^3^–10^4^ m^3^ and realistic magma compositions. Our results show importance of the deep degassing in the generation of volcanic seismicity and suggest that the DLP swarms beneath active volcanoes might be related to the pulses of volatile-rich basaltic magmas rising from the mantle.

## Abstract (web summary)

The origin of deep long-period earthquakes beneath active volcanoes that are sometimes considered as precursors to eruptions remains not fully understood. Here the authors show that these earthquakes can be generated by the rapid degassing in response to the slow decompression of magma over-saturated with H_2_O and CO_2_.

## Introduction

Deep Long Period (DLP) earthquakes occurring in middle to lower crust and uppermost mantle beneath volcanoes^1–9^ remain enigmatic and in some cases, are believed to have connection with magmatic activity. Similar to volcanic long-period (LP) seismicity in general^10^, the DLP earthquake has been considered to be generated by rapid pressure variations within magmatic plumbing systems. Alternatively, the effect of thermal stresses within cooling magma bodies has been considered^11^. The cooling magma stalled beneath the crust can also generate DLP earthquakes by so called “second boiling” or repeated pressurization of volatiles exsolved through crystallization, as has been recently suggested for dormant hot-spot Mauna Kea volcano in Hawaii^9^. However, such cooling-related mechanisms are unlikely for DLP events occurring beneath active volcanoes in association with eruptions. Different possible origins of pressure variations resulting in LP seismicity have been considered^12^ including the unsteady magma motion, breaking of mechanical “barriers”^9,13^, rapid degassing, etc. In any case, a reasonable model must provide a physical mechanism generating pressure variation *dP(t)* consistent with observed seismic waves. This implies that the time scale of these variations must by rather short, i.e., comparable with typical frequencies/periods of seismic waves (e.g., ~1 s). Second condition is that the fluid pressure variation should be strong enough and well coupled with the elastic media. This coupling may imply resonances of fluid-filled cracks or cavities^10^ that under certain conditions can result in nearly monochromatic and very long duration signals. At the same time, such “strongly resonant” features are not observed for DLP signals that are characterized by rather short durations.

**Fig. 1:**
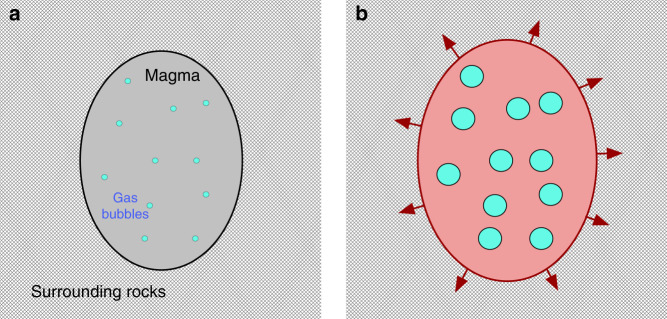
Conceptual model of fluid-related source of long-period earthquakes. **a** Bubble nucleation in a volume of magma saturated with H_2_O–CO_2_ volatiles. **b** Bubble and pressure growth deforming the surrounding rocks.

Here we propose that rapid changes of magmatic pressure near the crust-mantle boundary can be caused by nucleation and growth of gas bubbles in response to the slow decompression of over-saturated magma^14^. A volume of magma saturated with H_2_O–CO_2_ volatiles is subjected to slow de-pressurization because of its slow upwelling. This magma first reaches the saturation level and then achieves the critical supersaturation after which the gas bubbles nucleate (Fig. 1a) and grow very fast (Fig. 1b). Fast expansion of the bubbly magma deforms the surrounding rocks which respond elastically on the time scale associated with the bubble growth and magma pressure variations. As a result of this elastic rock deformation, seismic waves are radiated (Additional information provided in Methods) and can be recorded by seismographs installed in vicinity of volcanoes.

The pressure variation in the bubbly magma is simulated using the model that accounts for multiple dissolved volatiles (H_2_O–CO_2_) and diffusive gas transfer from magma into the growing bubbles. It is based on the full solution of advection-diffusion equation instead of quasi-static approach that was used before (Additional information provided in Methods)^15^. The bubble growth model is adopted to the case of bubble nucleation in basaltic magma^16^.

We compare the results of our modeling with DLP earthquakes observed beneath the Klyuchevskoy volcanic group (KVG) in Kamchatka, Russia. This volcanic group is one of the largest and most active clusters of subduction-zone volcanoes in the World^17^. KVG eruptions and their precursory periods are accompanied by sustained seismovolcanic activity including volcanic earthquakes^7,18,19^ and tremors^20^. We particularly focus on a persistent cluster of DLP earthquakes that occur in a small volume located at ~30 km depth beneath the Klyuchevskoy volcano^7,19,21^. The moment magnitudes (Additional information provided in Methods) of these DLP events range between 1.1 and 2.5 with maximum of their distribution at 1.4 (Supplementary Fig. 1).

Initial data on volatiles in Klyuchevskoy^22^ suggested that primary magmas content 2.2–2.9 wt.% of water. Later, a detailed study of melt inclusions in olivines^23^ has shown that parental magma has ~3.5 wt% H_2_O and 0.35–0.9 wt% CO_2_. Large increase of water content for some melt inclusions (up to 7 wt% H_2_O) was explained by de-compressional crystallization, accumulation volatiles in the melt phase and consequent slow degassing^23,24^. However, recent experimental data shows that the volatile content of Klyuchevskoy magma is much larger than the one previously directly measured in melt inclusions due to coupled SiO_2_-H_2_O loss^25^, suggesting that primary magma may contain more than 4 wt% of H_2_O. Single H_2_O volatile phase will result in a small saturation depth, but the addition of ~0.6 wt% of CO_2_ increases volatile solubility dramatically so that magma becomes supersaturated at pressures of 800 MPa (~30 km depth) that alternatively requires ~10 wt% of pure H_2_O.

We perform a parametric study to investigate the influence of volatiles content on the dynamics of bubble nucleation and growth. Our results show that the time scale of the bubble growth is mainly controlled by the gas and bubble content in the magma and under certain conditions can be sufficiently fast to generate seismic waves. In particular, we show that amplitudes and frequency content of DLP earthquakes observed beneath the Klyuchevskoy group of volcanoes can be predicted by our model when considering pressure changes of a few tens of MPa in a volume of ~10^3^–10^4^ m^3^ and magmas containing ~4 wt% of H_2_0 and ~0.6 wt% of CO_2_. Our results provide evidence for the role of the deep degassing in the generation of long-period volcanic seismicity and suggest that the DLP swarms observed beneath active volcanoes might be related to the pulses of fresh CO_2_–H_2_O rich basaltic magmas rising from the mantle.

## Results

### Volatiles content and the depth of degassing unset

**Fig. 2:**
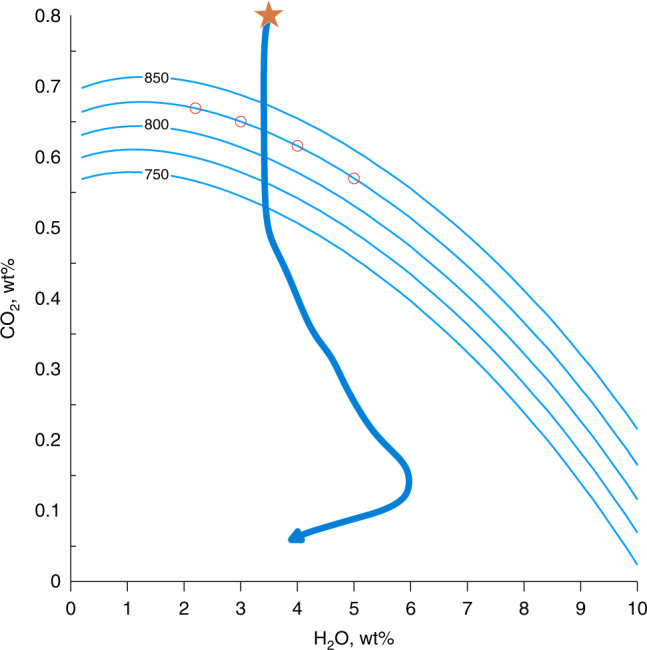
Gas saturation isobars as function of CO_2_–H_2_O content. Thin blue lines show saturation isobars for different pressures (indicated values in MPa). Thick solid line indicates the decompression path of the Klyuchevskoy magmas^24^ from initial state at 1 GPa shown with a star. Red circles show compositions along the 828 MPa isobar with 2, 3, 4, and 5 wt% of H_2_O tested with numerical modeling (results shown in Fig. 3).

Figure 2 shows how the solubility of the CO_2_–H_2_O mixture varies with pressure. Water and carbon dioxide concentrations were parametrized by polynomial functions of pressure and CO_2_ content in the bubble at a fixed temperature of 1230 ^o^C estimated from reversed crystallization of Klyuchevskoy melts from atmospheric conditions to 800 MPa pressure (Additional information provided in Methods) and extrapolated to 1000 MPa (Supplementary Table 1). The saturation point is reached at depths of the crust-mantle transition (~30 km; *P* ≈ 825 MPa) for magmas with the volatile content typical for the Klyuchevskoy volcano, implying that degassing may start at such large depths.

### Parameters controlling the time scale of bubble growth

**Fig. 3:**
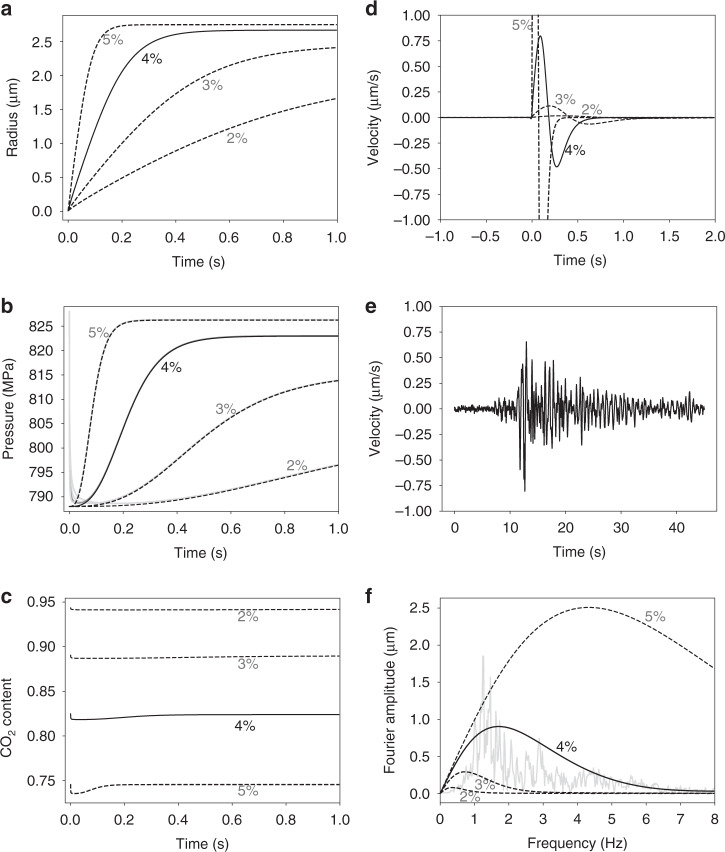
Modeled dynamics of the bubble grows and magma pressure change. Results are shown for the bubble number density of 10^13^ m^−3^, four different water contents indicated with wt% values in respective plots, and for CO_2_ content computed for 828 MPa (red circles in Fig. 2). **a** Evolution of the bubble radius. **b** Evolution of magma pressure *P*_*m*_ (*P*_*g*_ values are shown with gray lines). **c** Evolution of the CO_2_ content in bubbles. **d** Ground velocities estimated for a source located at a 30 km distance from the receiver (Additional information provided in Methods). **e** Example of real seismogram (east-west component at station LGN, Supplementary Fig. 1). **f** Fourier amplitudes computed from synthetic and real (gray line) signals.

Figure 3a, b shows typical evolution of bubble size, gas and melt pressure in the basaltic magma with density^26^ of 2800 kg m^−3^, viscosity of 10 Pa s and containing 10^13^ bubbles m^−3^ and for four different concentrations of H_2_O in initial magma. The CO_2_ contents (red circles in Fig. 2) were computed for initial pressure of 828 MPa that corresponds to the lithostatic pressure at depth of ~30 km for the average crustal density^27^ of 2830 kg m^−3^. Based on the experimental observations we adopt that the critical supersaturation for the bubble nucleation corresponds to the over-pressure of *ΔP* = 40 MPa^14,28^. This means that after nucleation the pressure in the gas bubble will be equal to its saturation value and the melt pressure is lower by *ΔP*. Due to rapid bubble expansion gas pressure decreases extremely fast while melt pressure starts to increase as the volume of the magma increases. Initially gas pressure drop due to bubble expansion dominates pressure increase due to volatile influx into the growing bubble. After reaching minimum value *P*_g_ starts to increase, concentration gradients in the melt become smoother and volatile flux decreases. At later stages of the growth the difference between melt and gas pressures becomes small and bubble growth is controlled by the diffusion of volatiles.

The water diffusion coefficient is 1–2 orders of magnitude larger than the diffusion coefficient for CO_2_. Thus, larger water content of magma for a fixed pressure require smaller amount of dissolved CO_2_ and bubble during growth will suck more H_2_O. Adding more water into initial magma results in H_2_O enriched gas and more vigorous bubble and pressure grows (Fig. 3). The effect of water content is enhanced even stronger in predicted seismograms (Fig. 3d) with H_2_O depleted magmas resulting in very weak signals. We compare amplitudes of synthetic seismograms computed for a magma source volume of 30,000 m^3^ (linear dimension of a few tens of meters) with a real seismogram (Fig. 3e) recorded during DLP earthquake with a magnitude *M*_W_ ≈ 2 at station LGN located nearly above the source region (Supplementary Fig. 1). Amplitudes and the frequency content (Fig. 3f) are reasonably well predicted with a model based on 4 wt% water in basaltic magma typical for the Klyuchevskoy volcanic group^25^.

We then perform a sensitivity study of several other parameters on the pressure evolution in the growing bubbles and resulting melt (Supplementary Fig. 2). Critical supersaturation^28^ that is required for bubble nucleation does not change melt-pressure recovery time significantly but will affect the amplitude of the source signal. We consider the melt viscosity range 10–10^5^ Pa s^29^. If viscosity is smaller than some threshold its influence on resulting pressure is negligible. Only larger melt viscosities typical for more silica reach melts (10^5^ Pa s) introduce some delay in pressure recovery. We assume instantaneous bubble nucleation in the whole batch of magma (Additional information provided in Methods). Thus, the size of the cell from which the bubble is growing is controlled by bubble number density (*BND*). We consider the *BND* range^30^ between 10^11^ and 10^15^ m^−3^. Increase in *BND* results in smaller cell sizes as *S*_0_ ~ *BND*^−1/3^. Melt pressure grows faster for smaller *S*_0_.

## Discussion

While the presented comparison of the observed and model-predicted seismograms (Fig. 3d–f) is based on significant simplifications of the source (ignoring realistic geometry and possible resonant behavior^10^) and the propagation effects (ignoring attenuation and wave scattering^31^), it shows that the amplitudes and the spectral content of the DLP signals observed at the Klyuchevskoy volcanic group can be explained to the order of magnitude by the bubble nucleation and growth in basaltic magmas according to the performed numerical simulation (Fig. 3d–f). Results of the presented modeling show that in the CO_2_–H_2_O rich basaltic magmas the degassing starts at large depths and is vigorous enough to produce strong and rapid pressure variations that can generate seismic radiation with amplitudes and frequency content comparable with those observed by seismographs during DLP earthquakes. Our results suggest that the DLP swarms observed beneath active volcanoes might be related to the intensification of the deep degassing caused by pulses of fresh CO_2_–H_2_O rich basaltic magmas rising from the mantle. This mechanism supports that the DLP earthquakes are early seismic manifestations of activation of deep parts of the Klyuchevskoy volcano plumbing systems. Similar behavior might be expected in other open and very active volcanic systems (with adjusting the model parameters based on their magma compositions and volatile contents). At the same time, magma-cooling-related DLP mechanisms can dominate beneath nearly closed or dormant volcanoes.

One of the key features of our model is that the depth of occurrence of DLP earthquakes is related to the CO_2_ content in magmas. This is especially interesting considering that global volcanic CO_2_ fluxes in modern Earth remain poorly known^32–38^ and are often estimated indirectly based on CO_2_/SO_2_ or other ratio proxies, with direct CO_2_ observations at volcanoes being technically challenging. Our results suggest that studies of the DLP volcanic seismicity provide additional constraints on the magmatic CO_2_ content in the deep roots of volcanoes.

## Methods

### Mathematical model of gas bubble growth

We consider growth of an individual bubble in the center of a spherical cell of melt that expands with the bubble and supports it with volatiles. The spherically symmetric model includes equations of mass conservation of the melt in a cell Eq. (1), diffusion equations for volatiles (H_2_O–CO_2_) Eq. (2), Rayleigh–Lamb equation for bubble growth with negligibly small inertia terms and the equation for the melt pressure evolution due to expansion of the surrounding elastic host rock Eq. (3), mass balances for volatiles in the bubble Eq. (4), and equations that describe physical properties of the components Eq. (5):

1 $$\frac{\partial }{{\partial r}}\left( {r^2\nu _r} \right) = 0;\left. {\nu _r} \right|_{r = R} \,=\, \frac{{dR}}{{dt}}$$

2 $$\frac{{\partial c_s}}{{\partial t}} + \nu _r\frac{{\partial c_s}}{{\partial r}} = \frac{1}{{r^2}}\frac{\partial }{{\partial r}}\left( {D_sr^2\frac{{\partial c_s}}{{\partial r}}} \right);s = CO_2(c),H_2O(w)$$

3 $$P_g - P_m = \frac{{2\sigma }}{R} + 4\mu \frac{{dR}}{{dt}}\left( {\frac{1}{R} - \frac{{R^2}}{{S^3}}} \right);P_m = P_m^0 + \frac{4}{3}G\left( {\frac{{S^3 - S_0^3}}{{S_0^3}}} \right)$$

4 $$\begin{array}{l}\frac{{4\pi }}{3}\frac{d}{{dt}}\left( {R^3\rho _gx_{CO2}^b} \right) = 4\pi R^2J_c;\frac{{4\pi }}{3}\frac{d}{{dt}}\left( {R^3\rho _g\left( {1 - x_{CO2}^b} \right)} \right) = 4\pi R^2J_w;\\ J_c = - D_c\rho _m\left( {\frac{{\partial c_c}}{{\partial r}}} \right)_{r = R};J_w = - D_w\rho _m\left( {\frac{{\partial c_w}}{{\partial r}}} \right)_{r = R}.\end{array}$$

5 $$\begin{array}{l}\rho _g = \left( {\frac{{x_{CO2}^b}}{{\rho _{CO2}\left( {P_g,T} \right)}} + \frac{{1 - x_{CO2}^b}}{{\rho _{H2O}\left( {P_g,T} \right)}}} \right)^{ - 1};\\ \rho _{CO2} = (0.371 + 0.13 \times 10^{ - 3}T) \cdot P_g + 1194.65 - 0.4665T;\\ \rho _{H2O} = (0.22 + 0.13 \times 10^{ - 3}T) \cdot P_g + 892.2 - 0.357T;\\ D_w = c_w \cdot {\mathrm{exp}}\left( { - 8.56 - \frac{{19110}}{T}} \right);\\ D_c = {\mathrm{exp}}\left( { - 13.99 - \frac{{(17367 + 1.945P_g)}}{T} + \frac{{c_w \cdot (855.2 + 0.271P_g)}}{T}} \right)\end{array}$$

Here *t* is time, *r* is the radial coordinate, *R* is the radius of the bubble, *v*_*r*_ is the radial velocity, *c*_*c*_ and *c*_*w*_ are the mass concentrations of CO_2_ and H_2_O in the melt, *D*_*c*_ and *D*_*w*_ are the volatile diffusion coefficients^39^, *P*_*g*_ is the pressure of the gas inside the bubble, *P*_*m*_ is the melt pressure, *σ* is the surface tension, *µ* is the magma viscosity, *S* is the radius of the cell, *G* is the shear modulus of the host rock, *ρ*_*g*_ is the density of the gas in the bubble that depends on the pressure, temperature *T* and bubble volatile composition $x_{CO2}^b$. The densities of pure CO_2_ (ρ_C02_)and H_2_O (ρ_H2O_) are approximated at a limited P-T range using tables produced by NIST Chemistry WebBook (https://webbook.nist.gov/chemistry/).

Equation (2) is subjected to two boundary conditions: concentration gradients are equal to zero at the outer surfaces of the cell mimicking symmetry of the system. At *r* = *R*(*t*) volatiles in magma are in chemical equilibrium with the bubble. Thus, $c_s = c_s^{eq}\left( {p,T,x_{CO2}^b} \right)$. We use D-compress software^40^ in order to calculate equilibrium concentrations.

The nucleation time of bubbles *t*_*n*_ from a supersaturated melt is related to the bubble number density *BND* via the nucleation rate *I*(m^−3^ s^−1^): *t*_*n*_ = *BND/I* According to classical nucleation theory^41^, *I* increases extremely fast with oversaturation pressure *ΔP:*$I\sim exp( - 1/{\mathrm{\Delta }}P^2)$. It depends on the temperature and a number of melt properties including surface tension, volume and concentration of water molecules in the melt, as well as distance between them, diffusion coefficient of volatiles at the bubble-melt interface, probability that a nucleus at the top of the barrier will go on to form the new phase, rather than dissolve (Zeldovich factor), and others. With a huge uncertainty of these parameters and difficulties in their experimental constrain, the estimated nucleation rate values vary by orders of magnitudes. For a basaltic melt with an overpressure about 40 MPa a value of *I~* 10^26^ m^−3^s^−1^ has been suggested^42^. With this *I*-value, nucleation time for *BND* = 10^13^ m^−3^ (values preferred in our study) is ~10^−13^ s, which is many orders of magnitude below the typical time scale of the simulated bubble growth and of the observed periods of seismic waves (~1 s). These estimations were obtained assuming that magma degassing is dominated by homogeneous nucleation. In the presence of crystals, their interfaces serve as a preferable location for the heterogeneous nucleation which takes roughly the same time, but produces significantly lower number of bubbles. In the case of heterogeneous nucleation, a pressure perturbation, induced by a limited number of new created bubbles, propagates through a magma-filled cavity providing a trigger for the homogeneous nucleation in the whole volume of magma. Such combination of heterogeneous and homogeneous nucleations is often assumed for many natural systems^28^. The duration of this process is controlled by a propagation time of a pressure pulse across a volume of over-saturated magma. With typical dimensions of a few tens of meters and sound speed being of the order of a few km/s, the combined heterogeneous and homogeneous nucleation will take less than 0.01 s, i.e., two orders of magnitude below typical bubble growth times. Therefore, we consider instantaneous nucleation in the whole volume.

### Numerical method

Equation (1) can be integrated analytically and gives the following velocity distribution in the melt phase: $v_r = \frac{{dR}}{{dt}}\frac{{R^2}}{{r^2}}$. In order to solve Eq. (2) in a fixed domain we use front-fixing method^43^. A coordinate transformation $\xi = \frac{{r - R\left( t \right)}}{{S\left( t \right) - R\left( t \right)}}$ gives extra advective term in Eq. (2). The resulting equation is discretized on an irregular 1D mesh with a decrease of the step size towards the growing bubble boundary (*ξ* = 0). The resulting system of equations with three-diagonal matrix is solved by means of Thomas algorithm^44^. The forward step starts at the outer domain boundary (*ξ* = 1). The linear relation of volatile concentration on the bubble boundary and in the nearest mesh points together with discretized Eqs. (3) and (4) allows to calculate all parameters on the bubble-melt interface. Then, concentration distribution in the whole domain is calculated during backward substitution. We found this method stable and computationally efficient in comparison with explicit methods that require extremely small timesteps for stability reasons.

### Estimation of magma composition

In order to estimate magma compositions in the deep magma reservoir we used “Petrolog” software^45^. Reverse crystallization from a more evolved magma (sample 12KY-108-1, 1987 AD eruption^46^) was performed. The starting pressure is set to atmospheric level and magma is H_2_O-saturated. Our simulations reveal total amount of mineral phase of 20% for the starting composition, which is in a good agreement with the measurements on the samples^47^. Incremental increase in pressure to 800 MPa leads to the change in composition presented in Supplementary Table 1. These values were obtained considering the volatile component composed only of H_2_O, resulting in a 800 MPa magma containing almost 11 wt% of dissolved water. Adding even a small amount of CO_2_ affects significantly the water solubility that can be reduced to a few wt% as shown in Fig. 2 along the 800 MPa isobar. Based on data about Klyuchevskoy magma volatile content^22–25,48^, we retain for our modeling a composition with ~4 wt% of H_2_0 and ~0.6 wt% of CO_2_.

### Estimation of magnitudes of deep low-frequency earthquakes

The DLP signals whose energy is concentrated in a narrow spectral band between 1 and 2 Hz are dominated by S-waves (Fig. 3e, f). The seismic moment can be approximately estimated from maximal signal amplitude in the following way. We start with an expression of the far-field (hypocenter distances exceeding 10 wavelengths) S-wave displacement^49^*u*^S^ and ignore the radiation pattern assuming that it approximately averages to 1. Based on this we can relate the time derivative of seismic moment with the observed S-wave displacement:

6 $$\dot M_0(t)\sim 4\pi \rho \beta ^3r \cdot u^{\mathrm{S}}(t)$$

where *t* is time *M*_0_ is seismic moment, *ρ* is density, *β* is S-wave speed, and *r* is the hypocentral distance. The observed ground velocity *v*^S^ is the derivative of the displacement that for a nearly monochromatic signal can be approximately estimated via multiplication by *2πf*^max^:

7 $$v^{\mathrm{S}}(t)\sim \dot u^{\mathrm{S}}(t)\sim 2\pi f^{{\mathrm{max}}} \cdot u^{\mathrm{S}}(t)$$

where *f*^max^ is the dominant signal frequency. Integration of Eq. (6) to obtain the whole seismic moment can be also approximately estimated with dividing by *2πf*^max^. This leads to a final expression used to approximately estimate the seismic moment from one station:

8 $$M_0\sim \frac{{4\pi \rho \beta ^3r \cdot u^{\mathrm{S}}}}{{2\pi f^{{\mathrm{max}}}}} = \frac{{\rho \beta ^3r}}{{\pi f^{{\mathrm{max}}2}}}\left| {v_{{\mathrm{max}}}^{\mathrm{s}}} \right|$$

where $v_{{\mathrm{max}}}^{\mathrm{s}}$ is the maximum amplitude of velocity seismograms (taking into account all three components). The final estimate is averaged from several stations that recorded the earthquake. We use *f*^max^ = 1.5 Hz and typical crustal values for density^27^, *ρ* = 2830 kg m^−3^, and seismic velocity^50,51^, *β* = 3500 m s^−1^. The moment magnitude *M*_*W*_ is then computed as:

9 $$M_{\mathrm{w}} = \frac{2}{3}\left( {\lg \left( {M_0} \right) - 9.05} \right)$$

### Estimation of seismic radiation emitted by expanding magma volume

For simplicity, we start with considering a volume with a perfectly spherical shape embedded in an infinite elastic space with bulk modulus *K*. In response to the magma pressure change *dP(t)*, the volume will be modified by *dV(t)*:

10 $$dV\left( t \right) = \frac{{dP\left( t \right)V}}{K}$$

For a perfectly spherical magma body, the volume change can be related to the seismic moment as^49^:

11 $$M_o\left( t \right) = KdV\left( t \right) = dP\left( t \right)V$$

A spherically symmetric source would radiate in the far field only P waves. At the same time, signals from real DLP earthquakes are dominated by S waves. A simple explanation of this observations can be related to the deviation of the magma body shape from a perfect sphere. In this case, the change of the magma pressure will induce a significant amount of shear stress in the surrounding rocks resulting in a strong S-wave radiation^52^. A possible example is a pure tensile crack mechanism for which the seismic moment tensor can be written as^49^:

12 $$M\left( t \right) = \left( {\begin{array}{*{20}{c}} {\lambda dV(t)} & 0 & 0 \\ 0 & {\lambda dV(t)} & 0 \\ 0 & 0 & {(\lambda + 2\mu )dV(t)} \end{array}} \right)$$

where *λ* and *μ* are Lamé constants that for most of elastic solids are nearly equal and have the same order of magnitude as bulk modulus (*K* = *λ* + 2/3*μ*) implying that to the order of magnitude the relationship (11) between seismic moment (observed amplitudes of waves), pressure variations, and volume of affected fluid remain valid. Seismic radiation from such source for many directions is dominated by S-waves^53^.

At this stage, we do not consider detailed description of seismic radiation from a non-spherical source that would vary significantly depending on the exact magma volume shape. We rather make an order of magnitude estimation and consider that Eq. (11) describes the relationship between the magma pressure change and the seismic moment observed in the far field (hypocenter distances exceeding 10 wavelengths). Based on Eq. (6), the ground displacement can be expressed as:

13 $$u\left( t \right) \approx \frac{{\dot M_0\left( t \right)}}{{4\pi \rho \beta ^3r}} = \frac{{\dot P\left( t \right)V}}{{4\pi \rho \beta ^3r}}$$

and the ground velocity is computed as its time derivative.

## Supplementary information

Supplementary Information

## Data Availability

The seismological time series used for the analysis were provided by the Kamchatka Branch of the Geophysical Survey of Russian Academy of Sciences (GS RAS) and are available on request (http://www.emsd.ru). The data are not publicly available due to the internal regulation of the GS RAS.

## References

[1] Pitt AM, Hill DP (1994). Long-period earthquakes in the Long-Valley Caldera region eastern California. Geophys. Res. Lett..

[2] White, R. A. in *Fire and mud: eruptions and lahars of Mount Pinatubo*, *Philippines* (eds Newhall, C. G. & Punongbayan, R. S.) 307–326 (Univ. Washington Press, 1996).

[3] Pitt AM, Hill DP, Walter SW, Johnston MJS (2002). Mid-crustal, long-period earthquakes beneath northern California volcanic areas. Seismol. Res. Lett..

[4] Power JA, Stihler SD, White RA, Moran SC (2004). Observations of deep long-period (DLP) seismic events beneath Aleutian arc volcanoes; 1989–2002. J. Volcanol. Geotherm. Res..

[5] Nichols ML, Malone SD, Moran SC, Thelen WA, Vidale JE (2011). Deep long-period earthquakes beneath Washington and Oregon volcanoes. J. Volcanol. Geotherm. Res..

[6] Aso N, Ohta K, Ide S (2013). Tectonic, volcanic, and semi-volcanic deep low-frequency earthquakes in western Japan. Tectonophysics.

[7] Shapiro NM (2017). Deep and shallow long-period volcanic seismicity linked by fluid-pressure transfer. Nat. Geosci..

[8] Hensch M (2019). Deep low-frequency earthquakes reveal ongoing magmatic recharge beneath Laacher See Volcano (Eifel, Germany). Geophys. J. Int.

[9] Wech AG, Thelen WA, Thomas AM (2020). Deep long-period earthquakes generated by second boiling beneath Mauna Kea volcano. Science.

[10] Chouet BA (1996). Long-period volcano seismicity: its source and use in eruption forecasting. Nature.

[11] Aso N, Tsai VC (2014). Cooling magma model for deep volcanic long-period earthquakes. J. Geophys. Res..

[12] Chouet BA, Matoza RS (2013). A multi-decadal view of seismic methods for detecting precursors of magma movement and eruption. J. Volcanol. Geotherm. Res..

[13] Shapiro NM, Campillo M, Kaminski E, Vilotte J‐P, Jaupart C (2018). Low‐frequency earthquakes and pore pressure transients in subduction zones. Geophys. Res. Lett..

[14] Lensky NG, Niebo RW, Holloway JR, Lyakhovsky V, Navon O (2006). Bubble nucleation as a trigger for xenolith entrapment in mantle melts. Earth Planet. Sci. Lett..

[15] Lyakhovsky V, Hurwitz S, Navon O (1996). Bubble growth in rhyolitic melts: experimental and numerical investigations. Bull. Volcanol..

[16] Gonnermann HM, Manga M (2005). Nonequilibrium magma degassing: results from modeling of the ca. 1340 AD eruption of Mono Craters, California. Earth Planet. Sci. Lett..

[17] Shapiro, N. M. et al. Understanding Kamchatka’s extraordinary volcano cluster. EOS 98, 10.1029/2017eo071351 (2017).

[18] Senyukov, S. L. *Forecasting of the eruptions of volcanoes Klyuchevskoy and Bezymianny at Kamchatka [in Russian]* (Lambert Academic, 2013).

[19] Senyukov SL (2009). Studies in the activity of Klyuchevskoi volcano by remote sensing techniques between January 1, 2001 and July 31, 2005, Volcanol. Seismol.

[20] Droznin DV (2015). Detecting and locating volcanic tremors on the Klyuchevskoy group of volcanoes (Kamchatka) based on correlations of continuous seismic records. Geophys. J. Int..

[21] Gorelchik VI, Garbuzova VT, Storcheus AV (2004). Deep-seated volcanic processes beneath Klyuchevskoi volcano as inferred from seismological data. J. Volcanol. Seismol..

[22] Khubunaya SA, Sobolev AV (1998). Primary melts of calc–alkaline magnesian basalts from Klyuchevskoy Volcano, Kamchatka, Dokl. Akad. Nauk.

[23] Portnyagin M, Hoernle K, Plechov P, Mironov N, Khubunaya S (2007). Constraints on mantle melting and composition and nature of slab components in volcanic arcs from volatiles (H_2_O, S, Cl, F) and trace elements in melt inclusions from the Kamchatka Arc. Earth Planet. Sci. Lett..

[24] Mironov NL, Portnyagin MV (2011). H_2_O and CO_2_ in parental magmas of Kliuchevskoi volcano inferred from study of melt and fluid inclusions in olivine. Russian Geol. Geophys..

[25] Portnyagin M (2019). Dehydration of melt inclusions in olivine and implications for the origin of silica-undersaturated island-arc melts. Earth Planet. Sci. Lett..

[26] Stolper E, Walker D (1980). Melt density and the average composition of basalt. Contrib. Mineral. Petrol..

[27] Christensen NI, Mooney WD (1995). Seismic velocity structure and composition of the continental crust: A global view. J. Geophys. Res. Solid Earth.

[28] Shea T (2017). Bubble nucleation in magmas: a dominantly heterogeneous process?. J. Volcanol. Geotherm. Res..

[29] Shaw HR (1972). Viscosities of magmatic silicate liquids; an empirical method of prediction. Am. J. Sci..

[30] Sable JE, Houghton BF, Del Carlo P, Coltelli M (2006). Changing conditions of magma ascent and fragmentation during the Etna 122 BC basaltic Plinian eruption: evidence from clast microtextures. J. Volcanol. Geotherm. Res..

[31] Aki K, Chouet B (1975). Origin of coda waves: source, attenuation, and scattering effects. J. Geophys. Res..

[32] Allard P (1991). Eruptive and diffuse emissions of CO_2_ from Mount Etna. Nature.

[33] Edmonds M (2008). New geochemical insights into volcanic degassing. Philos. Trans. R. Soc. A.

[34] Burton MR, Sawyer GM, Granieri D (2013). Deep carbon emissions from volcanoes. Rev. Mineral. Geochem..

[35] Hartley ME, Maclennan J, Edmonds M, Thordarson T (2014). Reconstructing the deep CO_2_ degassing ehavior of large basaltic fissure eruptions. Earth Planet. Sci. Lett..

[36] Kelemen PB, Manning CE (2015). Reevaluating carbon fluxes in subduction zones, what goes down, mostly comes up. Proc. Natl Acad. Sci. USA.

[37] Taran Y (2018). Gas emissions from volcanoes of the Kuril Island arc (NW Pacific): geochemistry and fluxes. Geochem. Geophys. Geosyst..

[38] Aiuppa A (2019). CO_2_ flux emissions from the Earth’s most actively degassing volcanoes, 2005–2015. Sci. Rep..

[39] Zhang Y., Ni H. (2010). Diffusion of H, C, and O Components in Silicate Melts. Reviews in Mineralogy and Geochemistry.

[40] Burgisser, A., Alletti, M. & Scaillet B. *D-Compress* https://vhub.org/resources/3791 (2015).

[41] Hirth G, Pound GM, St Pierre GR (1970). Bubble nucleation. Metall. Trans..

[42] Navon O, Lyakhovsky V (1998). Vesiculation processes in silicic magmas. Geol. Soc., Lond. Spec. Pub.

[43] Crank, J. *Free and moving boundary problems* (Oxford Science Publications, 1987).

[44] Mooney, D. D. & Swift, R. J. *A course in mathematical modeling* (Cambridge University Press, 1999).

[45] Danyushevsky LV, Plechov P (2011). Petrolog3: Integrated software for modeling crystallization processes. Geochem. Geophys. Geosyst..

[46] Bergal-Kuvikas O (2017). A petrological and geochemical study on time-series samples from Klyuchevskoy volcano, Kamchatka arc. Contrib. Mineral. Petrol..

[47] Bergal-Kuvikas, O. *Geochemical studies of volcanic rocks from the northern part of Kuril-Kamchatka arc: tectonic and structural constraints on the origin and evolution of arc magma*. Doctoral dissertation. P.190. (Hokkaido University, 2015).

[48] Auer S, Bindeman I, Wallace P, Ponomareva V, Portnyagin M (2009). The origin of hydrous, high-delta O-18 voluminous volcanism: diverse oxygen isotope values and high magmatic water contents within the volcanic record of Klyuchevskoi volcano, Kamchatka, Russia. Contrib. Mineral. Petrol..

[49] Aki, K. & Richards, P. G. *Quantitative seismology* (University Science Books, 2002).

[50] Levin V, Droznina S, Gavrilenko M, Carr MJ, Senyukov S (2014). Seismically active subcrustal magma source of the Klyuchevskoy volcano in Kamchatka, Russia. Geology.

[51] Droznina S (2017). S-wave velocity model for several regions of the Kamchatka Peninsula from the cross correlations of ambient seismic noise. Izvestiya Phys. Solid Earth.

[52] Eshelby JD (1959). The elastic field outside an ellipsoidal inclusion. Proc. R. Soc. A.

[53] Shi Z, Ben-Zion Y (2009). Seismic radiation from tensile and shear point dislocations between similar and dissimilar solids. Geophys. J. Int.

