# Supplementary material for: Deep long period volcanic earthquakes generated by degassing of volatile-rich basaltic magmas
Source: Nat Commun. 2020 Aug 6;11:3918. doi: 10.1038/s41467-020-17759-4 (PMC7414034; doi:10.1038/s41467-020-17759-4)
Supplement: Supplementary file 1 — Supplementary Information [file 41467_2020_17759_MOESM1_ESM.pdf]

Supplementary information

Degassing of volatile-rich basaltic magmas: source of deep long period volcanic earthquakes

O. Melnik, V. Lyakhovsky, N.M. Shapiro, N. Galina, and O. Bergal-Kuvikas

P (MPa)	T (^{o}C)	SiO_2	TiO_2	Al_2O_3	Fe_2O_3	FeO	MgO	CaO	Na_2O	K_2O	H_2O
0	1159.60	54.00	1.08	17.17	0.95	7.75	5.64	8.72	3.32	0.95	0.42
14	1161.20	53.45	1.07	16.99	0.94	7.68	5.61	8.63	3.28	0.94	1.41
38	1164.00	52.99	1.06	16.83	0.94	7.63	5.61	8.54	3.25	0.93	2.22
72	1167.90	52.52	1.05	16.66	0.94	7.57	5.62	8.46	3.22	0.92	3.04
113	1172.50	52.08	1.04	16.50	0.93	7.53	5.65	8.37	3.19	0.91	3.8
161	1178.00	51.64	1.03	16.33	0.93	7.49	5.69	8.29	3.16	0.90	4.54
213	1184.00	51.22	1.02	16.16	0.93	7.45	5.75	8.20	3.12	0.89	5.26
270	1190.40	50.82	1.01	16.00	0.93	7.42	5.81	8.12	3.09	0.88	5.92
330	1197.20	50.42	1.00	15.84	0.93	7.39	5.88	8.04	3.06	0.87	6.57
392	1204.20	50.03	0.99	15.68	0.94	7.36	5.95	7.96	3.03	0.87	7.19
457	1211.40	49.65	0.98	15.52	0.94	7.33	6.03	7.88	3.00	0.86	7.81
500	1216.30	49.40	0.97	15.41	0.94	7.31	6.08	7.82	2.98	0.85	8.24
800	1231.50	48.11	0.94	15.01	0.95	7.09	5.93	7.62	2.90	0.83	10.62

Supplementary Table 1. Evolution of the melt composition (in wt%) during reversed crystallization of Kluchevskoy magma.

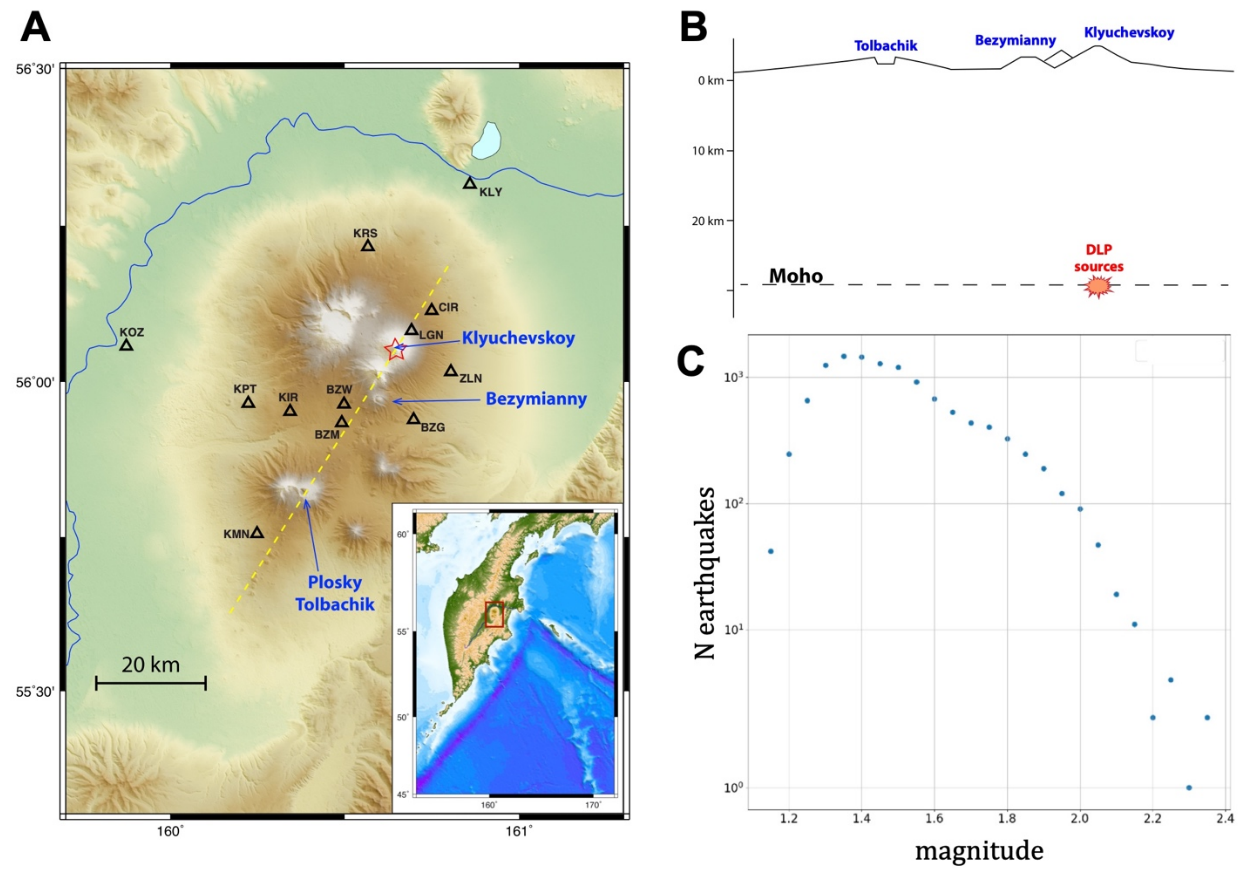

Supplementary Figure 1. Deep Long Period (DLP) earthquakes beneath the Klyuchevskoy volcanic group. (A) Map of the Klyuchevskoy volcano group with three active volcanoes indicated. Black triangles show stations of permanent seismic monitoring network. Red star shows approximate epicentral position of the DLP earthquake cluster. (B) Schematic vertical cross-section along the yellow dashed line shown in (A) indicating depth of the DLP earthquake cluster. (C) Event-size distribution (histogram) for one family of DLP multiplets.

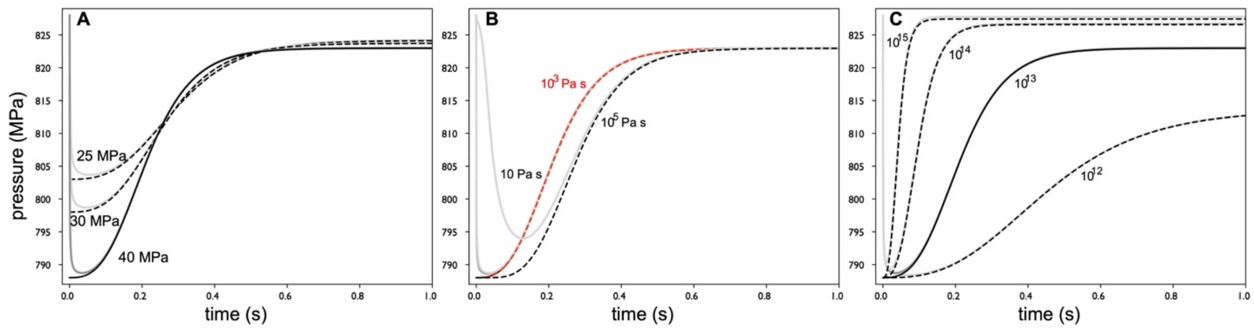

Supplementary Figure 2. Modeled gas and magma pressure changes. (A) Tests for varying pressure drop (μ = 10 Pa s; BND = 10^{13} m^{-3}; 4wt % H_20). (B) Tests for varying viscosity (ΔP = 40 Pa; BND = 10^{13} m^{-3}; 4 wt% H_20). (C) Tests for varying BND ((ΔP = 40 Pa; μ = 10 Pa s; 4 wt% H^20). Gas pressure is shown with grey lines.
